# Murine Cell Glycolipids Customization by Modular Expression of Glycosyltransferases

**DOI:** 10.1371/journal.pone.0064728

**Published:** 2013-06-14

**Authors:** Emili Cid, Miyako Yamamoto, Marcus Buschbeck, Fumiichiro Yamamoto

**Affiliations:** 1 The ABO Histo-blood Groups and Cancer Laboratory, Cancer Genetics and Epigenetics Program, Institut de Medicina Predictiva i Personalitzada del Càncer (IMPPC), Badalona, Catalunya, Spain; 2 The Chromatin and Cell Fate Laboratory, Cancer Genetics and Epigenetics Program, Institut de Medicina Predictiva i Personalitzada del Càncer (IMPPC), Badalona, Catalunya, Spain; Institut Curie, France

## Abstract

Functional analysis of glycolipids has been hampered by their complex nature and combinatorial expression in cells and tissues. We report an efficient and easy method to generate cells with specific glycolipids. In our proof of principle experiments we have demonstrated the customized expression of two relevant glycosphingolipids on murine fibroblasts, stage-specific embryonic antigen 3 (SSEA-3), a marker for stem cells, and Forssman glycolipid, a xenoantigen. Sets of genes encoding glycosyltansferases were transduced by viral infection followed by multi-color cell sorting based on coupled expression of fluorescent proteins.

## Introduction

Glycosphingolipids form the most widespread group of glycolipids in the animal kingdom. They are composed by one or more monosaccharides bound by a glycosidic linkage to ceramide. Glycosphingolipids are expressed ubiquitously in animal cells and are mainly present on the plasma membrane. They have been identified as highly relevant antigens that mark populations of cells such as cancer and stem cells [1]. Glycosphingolipids further act as receptors for microbial toxins and as modulators of cell adhesion, motility, and growth [2], and also bear blood group determinants, for instance, ABO [3]. Furthermore, they have been implicated in neural development [4], and in the pathogenesis of diseases such as neuropathies or glycosphingolipidoses [5].

However, the functional analysis of glycosphingolipids has been hampered by their complex nature and their combinatorial expression. Indeed, most of the cell membranes contain a mixture of different glycosphingolipids which can be subdivided in several different groups. Importantly, five of the most abundant groups have a common precursor, lactosylceramide (LC), which is galactosyl-β1→4-glucosyl-β1-ceramide.

Taking advantage of a cell line expressing LC as their major glycolipid we have devised a simple and streamlined methodology in order to create cells that homogenously and preferentially express specific glycosphingolipids.

## Materials and Methods

### cDNA, glycosyltransferase cloning and retroviral plasmid construction

Mouse Gbgt1 cDNA was obtained from Open Biosystems. Human A4GALT, B3GALNT1 and B3GALT5 cDNAs were cloned by RT-PCR from pooled total RNA from human cell lines (MCF-7, MDA-MB468, MDA-MB231, BT-20 and T-47D from ATCC; #HTB-22, HTB-132, HTB-26, HTB-19 and HTB-133 respectively) and normal human breast epithelial cells (Cambrex). First strand cDNA was synthesized using SuperScript III kit (Invitrogen). PCRs were performed using AccuPrime Pfx Supermix (Invitrogen). The oligonucleotide primers were custom synthesized (Invitrogen). Sense/antisense primers for each glycosyltransferase (GT) and TagFP open reading frame are the followings: A4GALT (TGCTGGAAGCTCCTGGTCTGATCT/CATCAGGAGCAGGTTGGG), B3GALNT1 (CTTCTGAGCTGCTGTGGATG/TCCTGTCCTTCTAGGCTTTT), B3GALT5 (TCAAGCTTATGGCTTTCCCGAAGATGAG/AACTCGAGTCAGACAGGCGGACAATCTT), Gbgt1 (CCGGAATTCCATGACCCGCCCAAGACTGGCCCAG/CCGGGATCCCTTAGGTCCTCAGCCAGTTGG), mTagBFP and TagRFP657 (ACCATGAGCGAGCTGATTAAGGAG/CGCTTTAATTAAGCTTGTGC). The GT cDNAs were cloned into pcDNA3.1-V5/His-TOPO vector (Invitrogen). The retroviral vector pMigR1 [6] was modified by swapping the EGFP coding sequence located after the internal ribosome entry sequence with mTagBFP2 (obtained by PCR using the vector pmTagBFP2-N1 [7], Addgene plasmid 34633) and TagRFP657 (from pTagRFP657-N1 [8]; Addgene plasmid 31959). Once the vectors were ready, the GT cDNAs were subcloned into the retroviral vector cloning site. DNA was obtained with the PureLink HiPrep midifilter kit (Invitrogen). All constructs were sequenced in order to avoid the introduction of artificial mutations.

### Packaging cell transfection

Phoenix-AMPHO cells [9] were seeded at a 2–3×10^6^ cells in 10 cm dish (Falcon) one day before transfection in DMEM (4.5 g/l d-glucose, 1 mM pyruvate, 2 mM glutamine) plus 10% Fetal Bovine Serum (FBS), penicillin (100 U/ml) and streptomycin (100 μg/ml) (Invitrogen).

Transfection was performed by mixing 20 μg of DNA, plus 61 μl of 2 M CaCl_2_ (Sigma) and brought to 500 μl with sterile culture-grade water. After a short vortex, 500 μl of 2× HEPES buffered saline (50 mM HEPES pH 7.05, 10 mM KCl, 12 mM d-glucose, 280 mM NaCl, 1.5 mM Na_2_HPO_4_ adjusted with NaOH to pH 7.05, Sigma) were added and the mixture was vortexed for 20 seconds, and incubated at room temperature for 5 minutes.

Meanwhile chloroquine (Sigma) was added to the Phoenix-AMPHO dish to a final concentration of 10 μM. After the incubation, the transfection mix was added drop by drop onto the cells; the dish was rocked and kept at the incubator for 8 hours (37°C, 5% CO_2_). The medium was replaced with fresh DMEM plus 10% FBS and antibiotics (8 ml).

### Cell infection with retroviral particles

L-M(TK-) cells [10] (ATCC# CCL-1.3) were seeded in 6-well plates at a 5×10^4^ cells/well density the day prior to infection.

The Phoenix-AMPHO medium was recovered with a syringe and filtered through a sterile 0.45 μm filter (Merck Millipore) into a centrifuge tube. 8 ml more of fresh medium were added and the cells were placed back into the incubator. Polybrene (Sigma) was added to a final concentration of 1 μg/ml to the filtered medium containing viral particles and 2 ml were added to the L-M(TK-) cells on the 6-well plate, and the plate was centrifuged at 750×g, for 45 min at 32°C. The medium containing the viral particles was removed and replaced with fresh medium. The cells were placed back in the incubator for 24 hours.

For each construct the infection was repeated 3 times using the same transfected Phoenix-AMPHO cells. In the case of pMigR1g-A4GALT cells were infected three times and then allowed to expand. These cells were checked by fluorescence for EGFP emission, and then were subjected to infection with viral particles from Phoenix-AMPHO cells transfected with pMigR1b-B3GALNT1 and pMigR1r-B3GALT5. In this case cells were infected consecutively, overlapping the third day of B3GALNT1 with the first of B3GALT5. Finally the infection with pMigR1r-Gbgt1 was done on previously sorted A4GALT/B3GALNT1 cells for three days.

### Fluorescence activated cell sorting

Once infections were finished, cells were allowed to grow to confluence in the 6-well plate and then detached with trypsin/EDTA (Invitrogen) and expanded into 10 cm dishes. When the dishes reached 90% confluency cells were detached again, spun down at 1,100×g for 3 min at room temperature, washed with phosphate buffered saline (pH 7.4, Sigma), spun again and resuspended with 3 ml of DMEM without phenol red (4.5 g/l d-glucose, 1 mM pyruvate, 2 mM glutamine) plus 2% FBS, penicillin (100 U/ml) and streptomycin (100 μg/ml). The cell suspension was sorted using a FACSAriaII (BD Biosciences) with a 4-way purity setting. Aggregates were excluded by selecting single cells by forward scatter-area versus forward scatter-height dot plots. FPs were excited and detected using a violet laser (405 nm) and a 450/40 band-pass filter for mTagBFP2, a blue laser (488 nm) and a 530/30 band-pass filter for EGFP and a red laser (640 nm) and a 660/20 band-pass filter for mTagRFP657. Background fluorescence and sorting gates were set for each population comparing them with non-transduced L-M(TK-) cells. Sorted cells were collected in DMEM supplemented with 20% FBS and streptomycin/penicillin and then plated onto 6 cm plates in DMEM plus 10% FBS. When confluent, they were detached with trypsin/EDTA and expanded into 10 cm dishes. Data were analyzed using FACSDiva software (BD Biosciences) and the final plots were generated by FlowJo (Tree Star).

### Immunocytochemistry

Cells were seeded at 2,000 cells/well on 8-cell Millicell EZ slides (Merck Millipore) for immunocytochemistry assays. Cells were allowed to reach around 60–90% confluence and then they were washed with PBS and fixed with 4% paraformaldehyde (Sigma) in PBS for 20 minutes at room temperature. They were rinsed with PBS and washed 3 times with PBS for 5 min. The wash to remove glycolipids was performed with 100% methanol (Fluka) for 15 min at room temperature, followed by PBS washes. Then, cells were blocked with diluted goat serum from ABC staining kit (Vector Lab) for 30 min. Antibodies were also diluted in this solution. Primary antibodies were rat IgM anti-SSEA-3 clone MC361 (Invitrogen) used at 1/100 dilution and rat IgM anti-Forssman antigen clone FOM-1 (BMA Biomedicals) at 1/500. Incubation was done at 4°C for 1 hour. Cells were washed again with PBS 4 times for 5 min. Secondary antibody was then applied, biotin-SP-conjugated AffiniPure goat anti-rat IgG+IgM (Jackson ImmunoResearch) at 1/1,000 dilution and incubated for 1 hour at 4°C. After a round of PBS washes, the immunostaining was amplified with avidin-biotin immunoperoxidase reagent (Vectastain ABC kit, Vector Lab) prepared as manufacturers’ specifications and incubated at room temperature for another hour. Then cells were washed with PBS and also with molecular grade water. Staining was performed by applying fresh diaminobenzidine (DAB) solution (Vector Lab) in water and the reaction was allowed to develop for 30–45 min at room temperature. DAB was removed and cells were washed with water and PBS. Controls were prepared by omitting the primary antibody incubation.

### Imaging and figure editing

Cells were imaged using a DMI6000B microscope equipped with color camera DFC420 and Leica Application Suite V3 software (Leica). Figures were created using Adobe Photoshop and Illustrator CS5 (Adobe).

## Results

With the purpose of creating customized glycosphingolipids we cloned glycosyltransferases (GTs) into retroviral vectors containing an internal ribosome entry sequence (IRES) element followed by different fluorescent proteins (FPs). Three different vectors [6] were used containing EGFP, mTagBFP [7] and TagRFP657 [8], respectively. These fluorescent proteins were chosen by their spectral properties to be easily excited and detected by the large majority of available cell sorters (equipped with violet, blue and red lasers). The presence of an IRES couples the GT of interest expression to the expression of a fluorescent protein allowing easy detection. Furthermore, integration into the host cell genome by using retroviral vectors permitted a stable expression of the construct, and the subsequent selection allowed generating homogeneously expressing cell populations.

**Figure 1:**
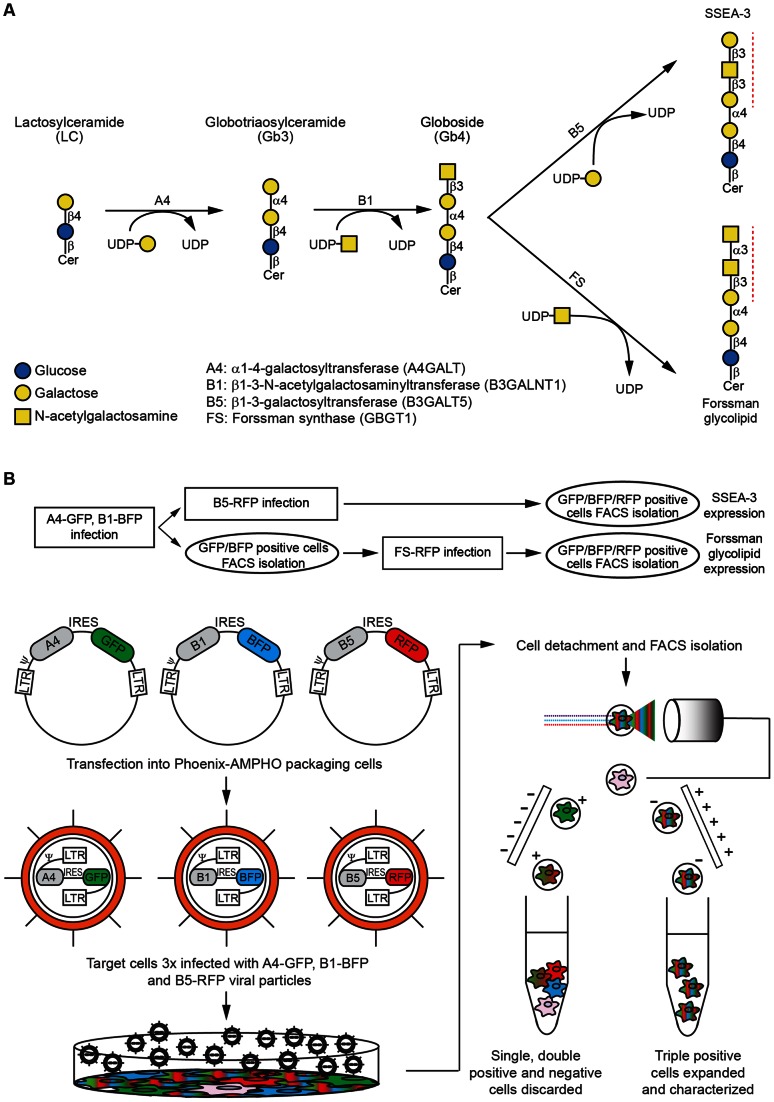
Schematics of the glycosphingolipids produced by L-M(TK-) cells and the methodology used. A. Biosynthetic pathways from lactosylceramide to SSEA-3 and Forssman antigens. The monosaccharides are color and shape coded as the legend details. UDP is uridine diphosphate and Cer is ceramide. Glycosyltransferase activities are shown on top of the arrows and red dashed lines indicate the epitopes recognized by the antibodies used. The right legend describes the abbreviation used throughout the article, the enzyme names and, between parentheses, the gene symbols. Glycosidic bonds are represented in a compact form (i.e. β4 standing for β 1→4). B. Flow chart and diagram explaining the protocol. Briefly, retroviral constructs containing glycosyltransferase cDNAs, an IRES and a fluorescent protein are transfected into packaging cells. The resulting viral particles are used to infect L-M(TK-) cells. After expansion, cells were detached from culture dishes. In the case of SSEA-3 expressing cells, cells were infected sequentially with all three viral particles (A4-GFP, B1-BFP and B5-RFP) and then triple positive cells were FACS sorted as shown schematically in the diagram. For Forssman antigen expressing cells, after infection with A4-GFP and B1-BFP viral particles, the double positive cells were FACS sorted and expanded first. Then they were infected with FS-RFP viral particles and finally the triple positive cells were isolated after a second FACS procedure as detailed in the flow chart.

As proof of principle, we focused on two well known glycosphingolipids, the stage specific embryonic antigen-3 (Galβ1→3GalNAcβ1→3Galα1→4Galβ1→4Glcβ1→ceramide) (SSEA-3) and the Forssman glycolipid (GalNAcα1→3GalNAcβ1→3Galα1→4Galβ1→4Glcβ1→ceramide). The biosynthetic pathways leading to these two molecules from LC are shown in Figure 1 A and involve two shared enzymes α-1,4-galactosyltransferase (A4) and β-1,3-N-acetylgalactosaminyltransferase 1 (B1) followed by the unique action of GlcNAc/GalNAc-β-1,3-galactosyltransferase 5 (B5) [11] and Forssman synthase (FS) in case of SSEA-3 and Forssman antigen, respectively. We generated retroviral vectors with the cDNAs of all required GTs. The constructs information consisting of the vector used, its corresponding fluorescent protein, the GT name, gene symbol and abbreviation used throughout the paper is summarized in Table 1.

**Table 1:** Summary of the retroviral constructs used.

Abbreviation	Gene symbol	Enzyme name	Vector (fluorescent protein)
A4	A4GALT	α1,4-galactosyltransferase	pMigR1g (EGFP)
B1	B3GALNT1	β1,3-N-acetyl-galactosaminyltransferase 1	pMigR1b (mTagBFP)
B5	B3GALT5	GlcNAc/GalNAc-β1,3-galactosyltransferase 5	pMigR1r (TagRFP657)
FS	Gbgt1	Forssman synthase	pMigR1r (TagRFP657)

Each enzyme corresponding to a gene symbol was abbreviated in the text and its cDNA introduced into retroviral vectors containing a different fluorescent protein (between parentheses).

Viral particles were generated by transfection of a packaging cell line using standard protocols [12]. The amphotropic HEK293 based Phoenix turned out to be most suitable host cells for preparing the viral particles to transduce L-M(TK-) cells.

Next, we sequentially infected L-M(TK-) cells with A4 and B1 particles to obtain the common precursor of SSEA-3 and Forssman antigen. At this point, cells were either sorted to isolate double positive cells or expanded and transduced with the vector encoding the third GT. They were analyzed and sorted by FACS. A schematic diagram of the protocol is shown in Figure 1 B.

**Figure 2:**
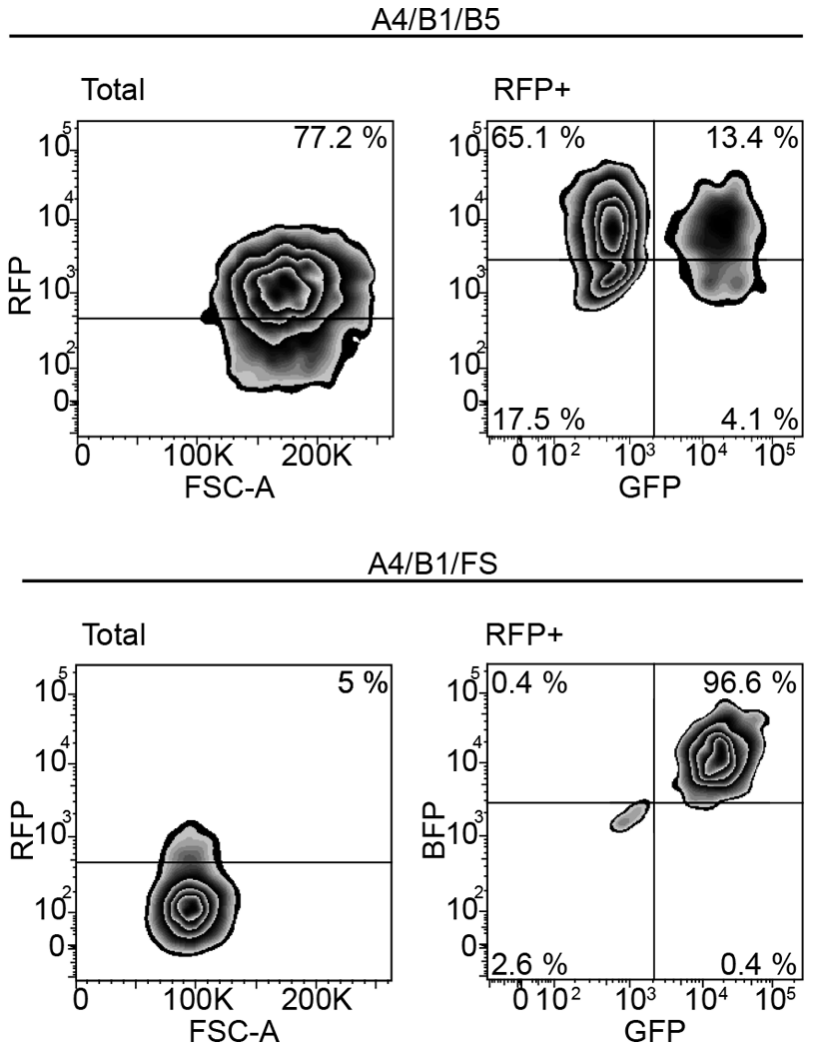
Fluorescence Activated Cell Sorting of triple positive cells. Cytometry analysis of the triple positive cells to be sorted. The Total zebra plot depicts the RFP fluorescence in logarithmic scale versus the forward scattering and on top of the threshold, the percentage of positive cells. The RFP+ plot shows the blue and the green fluorescence from the RFP positive cells only, thus the right upper population represents the triple positive cells.

The results of the cytometry analysis are shown in Figure 2. In the case of cells geared to SSEA-3 synthesis, although the percentage of cells positive for the individual FPs varied (up to 85%), a sufficient number of cells was positive for RFP (77.2%) and the triple positive reached a 13.4% and could be isolated without any previous interim sorting (147,848 cells). Presorting of double positive A4+B1+ cells to >95% purity allowed us to gain a sufficient number of triple positive cells containing also FS even though we detected only a little more than 5% of RFP positive cells. Again we used triple color sorting to extract the triple positive cell population of interest (51,079 cells).

**Figure 3:**
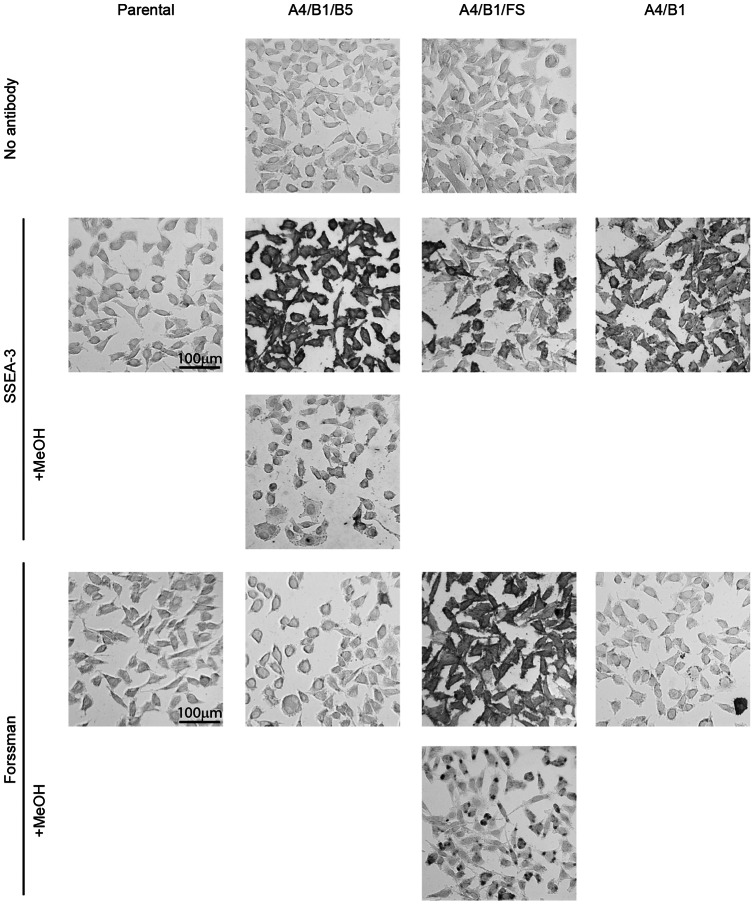
Glycan immunocytochemistry of triple positive cells geared towards the synthesis of SSEA-3 and Forssman antigens. DAB staining using the ABC immunocytochemical system of sorted triple positive cells. Genotypes are described in the horizontal axis while the antibodies and staining procedures are organized on the vertical axis. The “No antibody” row corresponds to staining only with secondary antibody. The SSEA-3 staining was performed using the MC361 antibody. The Forssman antigen was detected with FOM-1 antibody. All images were taken at the same magnification.

After sorting, and to evaluate the validity of the method, we seeded glass cell chambers with triple positive cells, the double expressing cell controls and unmodified L-M(TK-) cells and fixed them with paraformaldehyde to perform immunocytochemistry. We incubated the cells with commercially available antibodies against these antigens, followed by a secondary antibody conjugated to biotin incubation and finally by avidin-biotin peroxidase ABC reagent. In order to develop the stain we utilized DAB solution. As it can be seen in Figure 3, triple positive cells not incubated with primary antibodies and parental L-M(TK-) cells treated with both primary antibodies do not show any signal. On the other hand, triple positive cells are strongly stained indicating a homogeneous and abundant presence of both SSEA-3 (A4/B1/B5 cells) and Forssman (A4/B1/FS cells) antigens. In order to exclude possible cross-reactivities with protein conjugates we have pretreated our samples with methanol, which is known to efficiently remove glycosphingolipids while not affecting glycoprotein or proteoglycans staining on paraformaldehyde fixed cells [13]. As further shown in Figure 3, methanol pretreatments eliminated virtually all staining of SSEA-3, and only left a small amount of staining for Forssman antigen, possibly representing the minor intracellular fraction.

In the case of A4/B1 cells there is some SSEA-3 staining but at lower levels when compared to the triple positive population. This staining could be due to weak recognition of the globoside precursor by the anti SSEA-3 antibody used [14] although it does not exclude the possibility of a low level of endogenous β3-galactosyltransferase activity onto the globoside created by the introduction of the other two enzymes.

When using anti-Forssman antibody FOM-1, there is a very small percentage, around 2%, of stained cells in the A4/B1 and A4/B1/B5 populations indicating that a very small fraction of cells might be expressing FS endogenously. We would also like to highlight the fact that A4/B1/FS cells show less positive cells for SSEA-3 than A4/B1, probably because of the competitive use of globoside by FS. All these data indicates that we are able to create homogeneous cell populations that present specific glycosphingolipids at the membrane.

## Discussion

One of the advantages of this method is the use of a fibroblastic type cell of small size. L-M(TK-) cells are easily detached, not forming clumps and with a homogenous shape that facilitates cell sorting. The length of most GT cDNAs is well within the packaging capacity of retroviral particles and, on top of that, L-M(TK-) cells are readily and efficiently transduced with these vectors. In our hands, the expression of the introduced heterologous GTs and FPs genes is stable, but if it was necessary, cell lines could be created by clonal expansion of cells expressing high levels of antigen. Once the constructs are created, a simple, systematic and streamlined protocol within a medium throughput context will deliver populations of cells with a stable and homogenous expression of any glycolipid of interest for follow-up studies.

This approach can be expanded to other fluorescent markers thus further increasing the combinatorial spectrum of the approach while avoiding expensive multiple drug selections. The human genome encodes a large number of GTs giving rise to a large number of different glycolipids. At least 341 different ones build on LC have been described [15]. One of the key characteristics of glycan moieties is their indirect biosynthesis through enzymatic reactions when compared to proteins, which are encoded directly by genetic material. This means that availability of substrate and competing reactions have a great impact on the production of a specific carbohydrate chain. Therefore, overexpression of a glycoslytransferase will not only favor the synthesis of its product, but also diminish the presence of molecules that are produced from the same substrates. Moreover, the stepwise nature of oligosaccharides prevents the action of enzymes unless their immediate substrate is present. Complementarily, reducing the expression of key GTs would decrease the presence of a large set of glycolipids whose synthesis depends on the catalysis of a common step. Therefore, another possibility would be the modulation of glycosphingolipids in any cell-type by downregulation of these enzymes by RNA interference. This methodology preceded by a GT expression profiling might help in the study of glycosphingolipid molecular functions: interactions with receptors, effects on cell morphology, attachment or drug sensitivity, just to cite some examples.

A direct application of a library created by our here described approach with L-M(-TK) cells is antibody production. Many of the monoclonal antibodies against glycans antigens currently used were created using cells and tissues that present these antigens on the surface, even though their glycan landscape is probably more complex than the one these engineered cells have. The glycolipid antigens have to be obtained by lipid extraction followed by purification strategies or by enzymatic/chemical synthesis approaches. By introducing GTs in a hierarchal mode, a great amount of glycan antigens could be generated within a versatile cellular framework. We have just embarked on the creation of this cell library to screen antibody phage display libraries in order to obtain novel antibodies against known and unknown glycan antigens.

## References

[1] Hakomori SI (2002) Glycosylation defining cancer malignancy: new wine in an old bottle. Proc Natl Acad Sci U S A 99: 10231–10233. 12149519 10.1073/pnas.172380699 PMC124893

[2] Regina Todeschini A, Hakomori SI (2008) Functional role of glycosphingolipids and gangliosides in control of cell adhesion, motility, and growth, through glycosynaptic microdomains. Biochim Biophys Acta 1780: 421–433. 17991443 10.1016/j.bbagen.2007.10.008 PMC2312458

[3] Yamamoto F, Cid E, Yamamoto M, Blancher A (2012) ABO research in the modern era of genomics. Transfus Med Rev 26: 103–118. 21945157 10.1016/j.tmrv.2011.08.002

[4] Yu RK, Tsai YT, Ariga T (2012) Functional roles of gangliosides in neurodevelopment: an overview of recent advances. Neurochem Res 37: 1230–1244. 22410735 10.1007/s11064-012-0744-y PMC3895947

[5] Zhang X, Kiechle FL (2004) Review: Glycosphingolipids in health and disease. Ann Clin Lab Sci 34: 3–13. 15038664

[6] Pear WS, Miller JP, Xu L, Pui JC, Soffer B, et al (1998) Efficient and rapid induction of a chronic myelogenous leukemia-like myeloproliferative disease in mice receiving P210 bcr/abl-transduced bone marrow. Blood 92: 3780–3792. 9808572

[7] Subach OM, Cranfill PJ, Davidson MW, Verkhusha VV (2011) An enhanced monomeric blue fluorescent protein with the high chemical stability of the chromophore. PLoS One 6: e28674. 22174863 10.1371/journal.pone.0028674 PMC3234270

[8] Morozova KS, Piatkevich KD, Gould TJ, Zhang J, Bewersdorf J, et al (2010) Far-red fluorescent protein excitable with red lasers for flow cytometry and superresolution STED nanoscopy. Biophys J 99: L13–15. 20643047 10.1016/j.bpj.2010.04.025 PMC2905082

[9] Swift S, Lorens J, Achacoso P, Nolan GP (2001) Rapid production of retroviruses for efficient gene delivery to mammalian cells using 293T cell-based systems. Curr Protoc Immunol Chapter 10: Unit 10 17C. 10.1002/0471142735.im1017cs31 18432682

[10] Kit S, Dubbs DR, Piekarski LJ, Hsu TC (1963) Deletion of thymidine kinase activity from L cells resistant to bromodeoxyuridine. Exp Cell Res 31: 297–312. 14056989 10.1016/0014-4827(63)90007-7

[11] Zhou D, Henion TR, Jungalwala FB, Berger EG, Hennet T (2000) The beta 1,3-galactosyltransferase beta 3GalT-V is a stage-specific embryonic antigen-3 (SSEA-3) synthase. J Biol Chem 275: 22631–22634. 10837462 10.1074/jbc.C000263200

[12] Buschbeck M, Hofbauer S, Di Croce L, Keri G, Ullrich A (2005) Abl-kinase-sensitive levels of ERK5 and its intrinsic basal activity contribute to leukaemia cell survival. EMBO Rep 6: 63–69. 15608616 10.1038/sj.embor.7400316 PMC1299226

[13] Yanagisawa M, Yoshimura S, Yu RK (2011) Expression of GD2 and GD3 gangliosides in human embryonic neural stem cells. ASN Neuro 3: 69–74. 10.1042/AN20110006 PMC3072763 21395555

[14] Kannagi R, Cochran NA, Ishigami F, Hakomori SI, Andrews PW, et al (1983) Stage-specific embryonic antigens (SSEA-3 and −4) are epitopes of a unique globo-series ganglioside isolated from human teratocarcinoma cells. Embo J 2: 2355–2361. 6141938 10.1002/j.1460-2075.1983.tb01746.x PMC555457

[15] Yu RK, Yanagisawa M, Ariga T (2007) Glycosphingolipid Structures. In: Kamerling JP, editor. Comprehensive Glycoscience: From Chemistry to Systems Biology. Amsterdam: Elsevier Science.

